# Supplementary material for: Deciphering cell-type-and temporally specific matrisome expression signatures in human cortical development and neurodevelopmental disorders via scRNA-seq meta-analysis
Source: Nat Commun. 2025 Nov 11;16:9907. doi: 10.1038/s41467-025-64381-3 (PMC12606316; doi:10.1038/s41467-025-64381-3)
Supplement: Supplementary file 1 — Supplementary Information [file 41467_2025_64381_MOESM1_ESM.pdf]

# Supplementary Information

## Deciphering Cell-Type and Temporal-Specific Matrisome Expression Signatures in Human Cortical Development and Neurodevelopmental Disorders via scRNA-Seq Meta-Analysis

Do Hyeon Gim[1], Muhammad Z.K. Assir[1], Olivia Soper[1], Paul A. Fowler[1], Michael D. Morgan[1], Daniel A. Berg[1, *], Eunchai Kang[1,*]

[1] Institute of Medical Sciences, School of Medicine, Medical Sciences and Nutrition, University of Aberdeen

Corresponding author: Eunchai Kang, Daniel A. Berg

**The PDF file includes:**

Supplementary Figures 1 to 6

**Other Supplementary Information for this manuscript includes the following:**

Supplementary Data 1 to 14

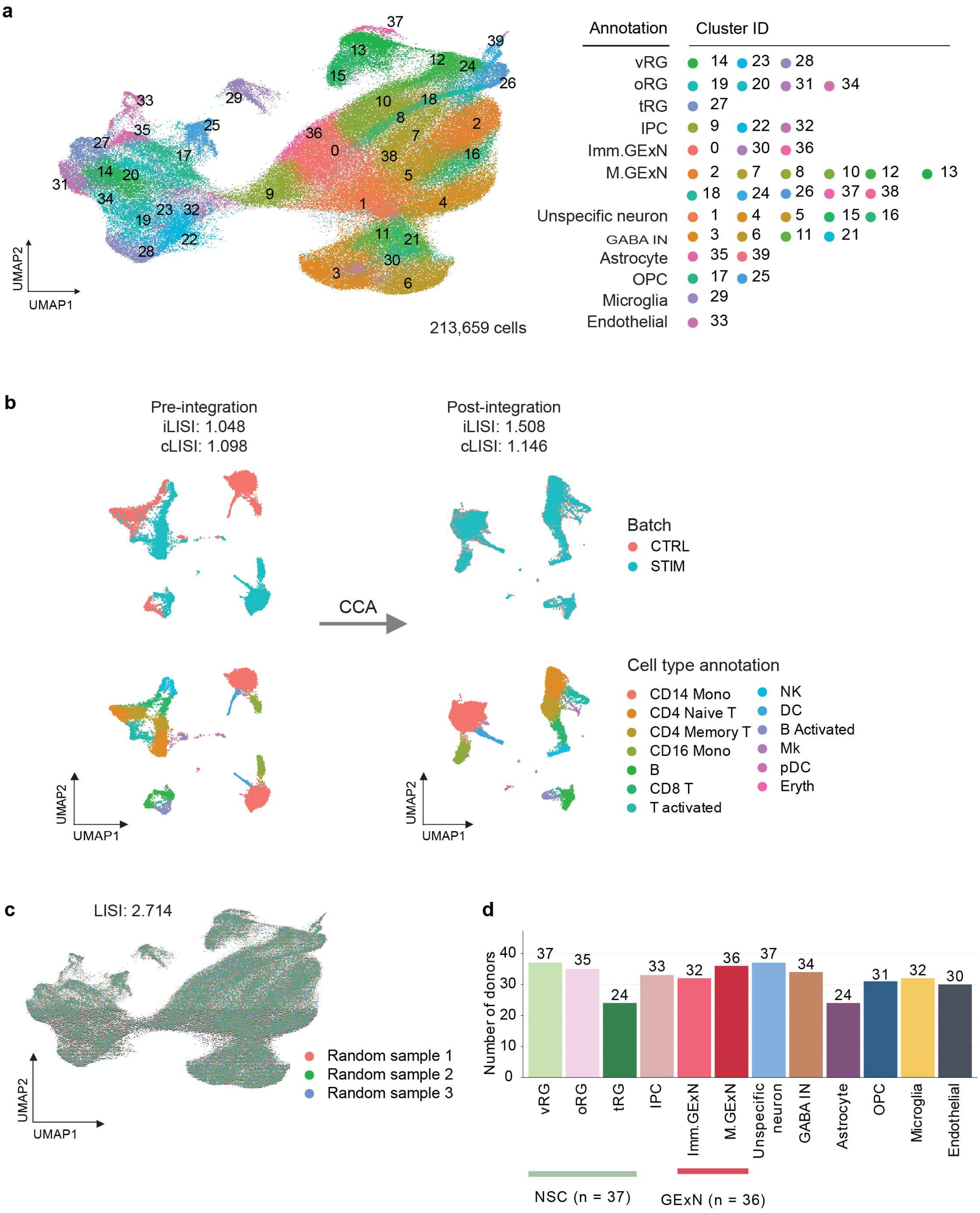


**Supplementary Fig. 1: Cell type annotation of meta-data and validation of integration**

**a** Unsupervised cell type annotation: 40 clusters were identified in the meta-data, and each cluster was annotated based on cell type using the scType pipeline (left). A table presents the corresponding cell type for each cluster ID (right).

**b** UMAP visualization of two groups of peripheral blood mononuclear cell (PBMC) data—control (CTRL) and interferon beta-stimulated (STIM)—before and after CCA integration. The Integration Local Inverse Simpson's Index (iLISI) and Cell-Type LISI (cLISI) scores are displayed before and after integration. Notably, iLISI increased from 1.048 to 1.508 following integration.

**c** UMAP visualization of the scRNA-seq meta-data with random sampling annotations (Sample 1–3) representing an ideal batch integration. The Local Inverse Simpson's Index (LISI) for perfect batch mixing is shown as the maximum score reference for this dataset.

**d** Bar graph displaying the number of donors per cell type.

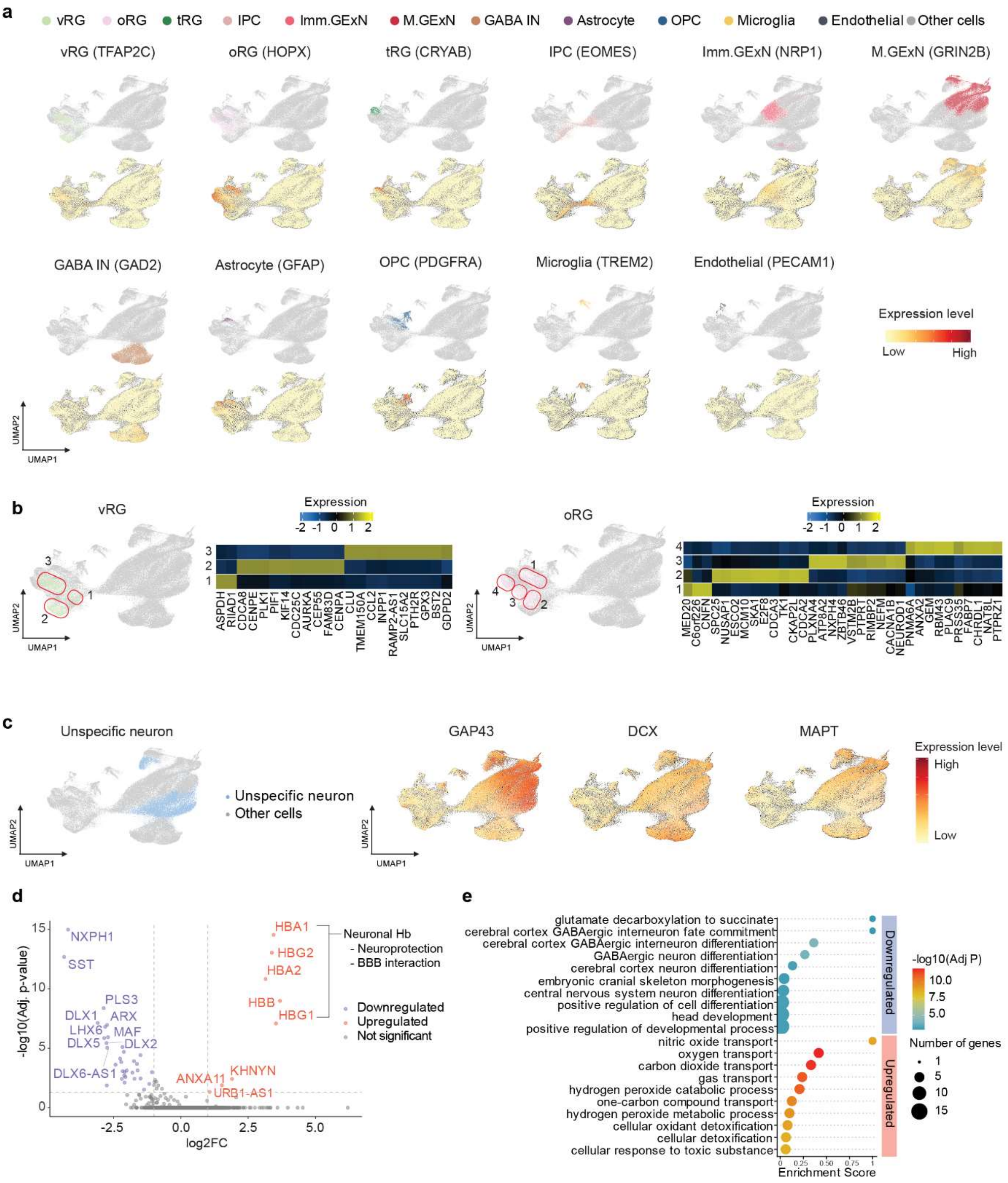


**Supplementary Figure 2: Validation and Characterization of Cell Type Annotation**

**a** UMAP visualization of the scRNA-seq meta-data highlighting all cell type annotations and known cell type marker genes. Expression levels of marker genes are shown on a scaled color gradient.

**b** UMAP visualization of the scRNA-seq meta-data highlighting distinct vRG clusters (left) and oRG clusters (right). Red circles indicate each cluster of cells, labeled with cluster numbers. A heatmap displays differentially expressed genes between the clusters. The heatmap represents DEG expression levels across distinct clusters, where each column corresponds to a gene, and each row represents a cell type ($\log_2$ fold-change > 1, adjusted $p$-value < 0.05).
**c** UMAP of scRNA-seq meta-data highlighting unspecific neuron and expression of neuronal markers, *GAP43*, *DCX* and *MAPT*.
**d** Volcano plot showing differentially expressed genes (DEGs) of unspecific neuron in comparison to GExN and GABA IN. Most upregulated genes in unspecific neuron are neuronal haemoglobin (Hb) genes.
**e** Gene ontology (GO) term analysis of DEGs of unspecific neuron. The x-axis represents an enrichment score. The size of dots represents number of genes in the annotation and $-\log_{10}$ adjusted $p$-value is represented in color scale.

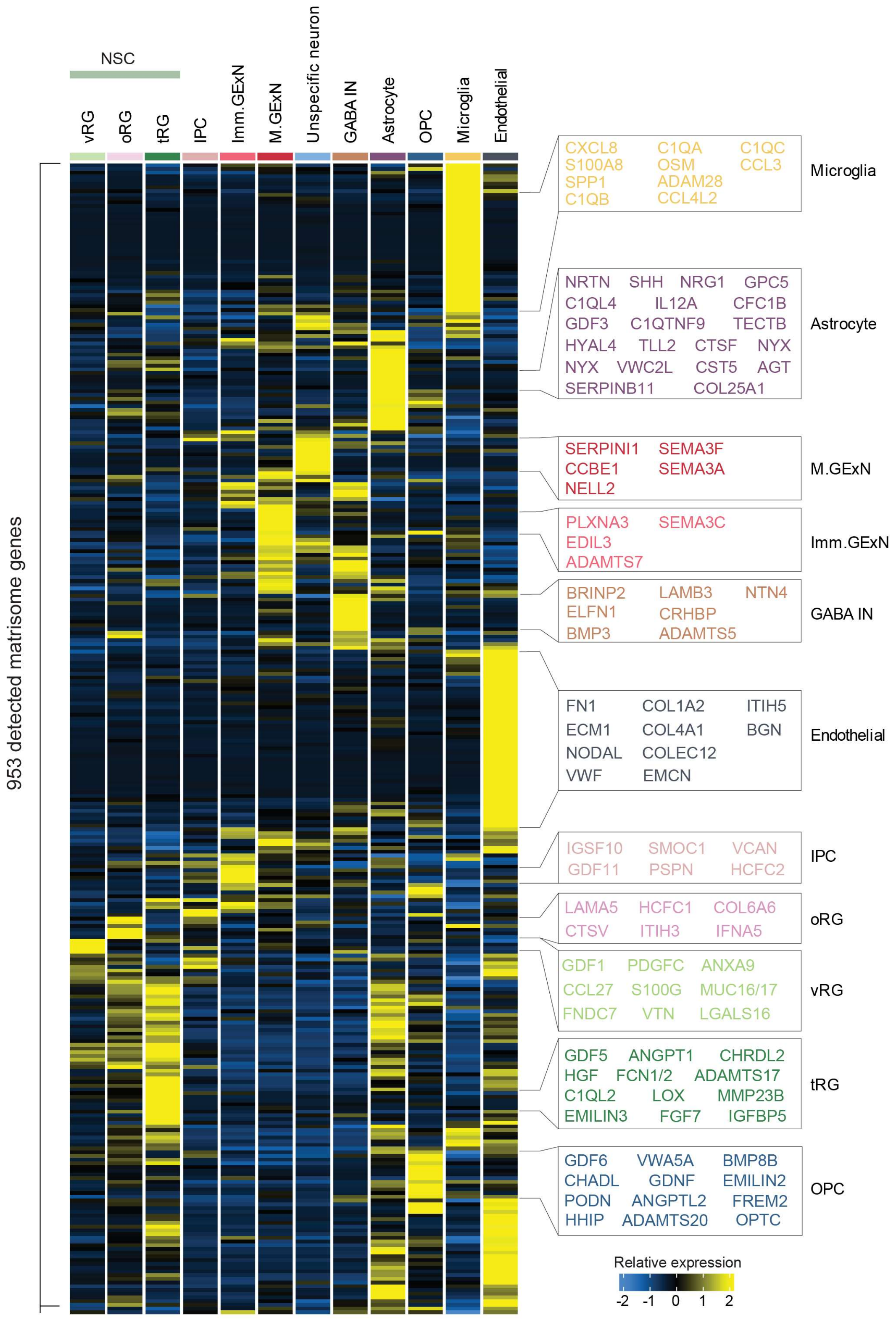


**Supplementary Fig. 3: Matrisome expression across the various cell types**

Heatmap of relative expression levels of 953 matrisome genes across 12 cell types. Each row represents a gene, and each column corresponds to a cell type, highlighting cell type-specific signatures of matrisome gene.

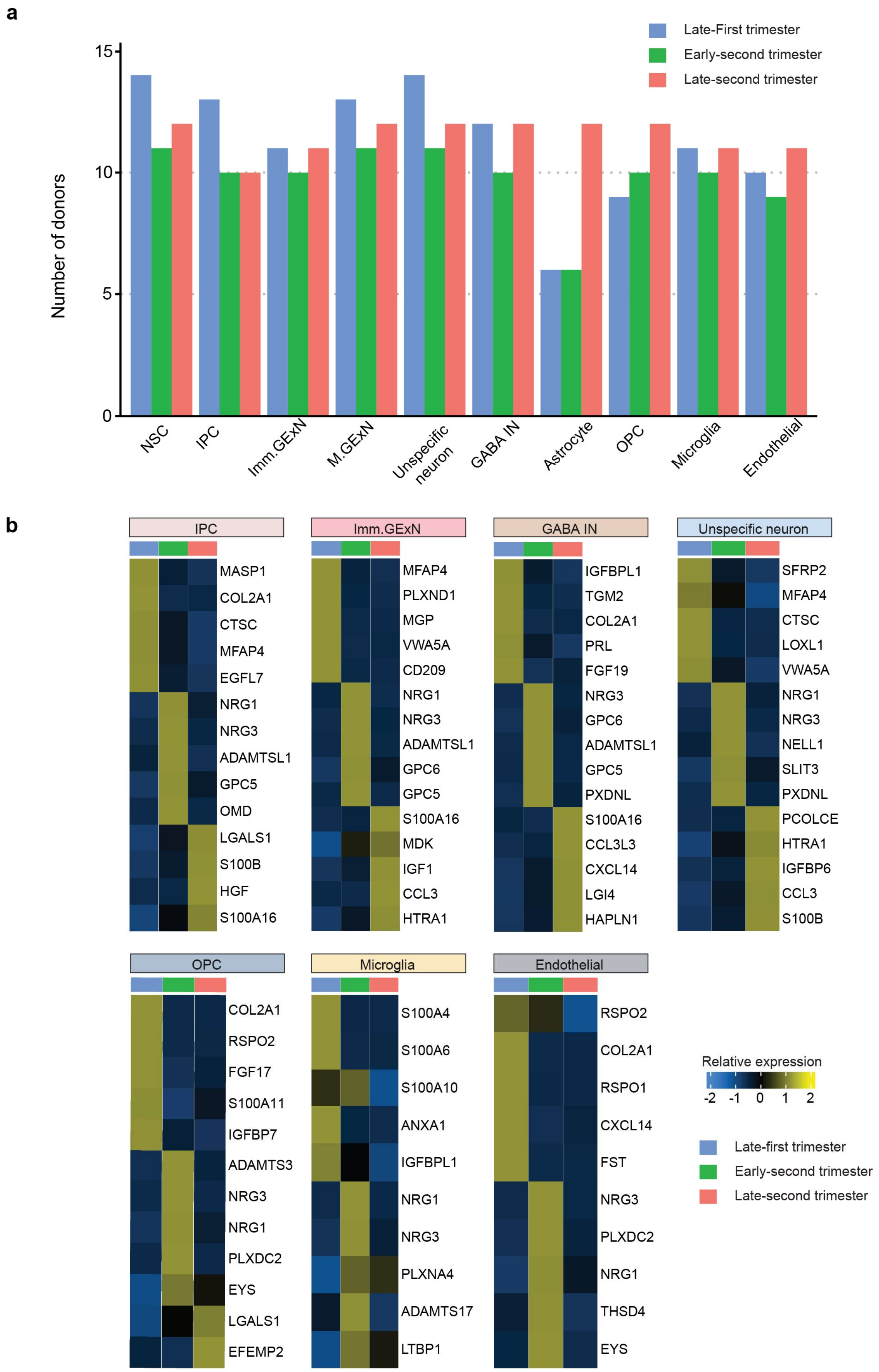
a
Late-First trimester
Early-second trimester
Late-second trimester
Number of donors
0
5
10
15
NSC
IPC
Imm.GExN
M.GExN
Unspecific neuron
GABA IN
Astrocyte
OPC
Microglia
Endothelial
b
IPC
MASP1
COL2A1
CTSC
MFAP4
EGFL7
NRG1
NRG3
ADAMTSL1
GPC5
OMD
LGALS1
S100B
HGF
S100A16
Imm.GExN
MFAP4
PLXND1
MGP
VWA5A
CD209
NRG1
NRG3
ADAMTSL1
GPC6
GPC5
S100A16
MDK
IGF1
CCL3
HTRA1
GABA IN
IGFBPL1
TGM2
COL2A1
PRL
FGF19
NRG3
GPC6
ADAMTSL1
GPC5
PXDNL
S100A16
CCL3L3
CXCL14
LGI4
HAPLN1
Unspecific neuron
SFRP2
MFAP4
CTSC
LOXL1
VWA5A
NRG1
NRG3
NELL1
SLIT3
PXDNL
PCOLCE
HTRA1
IGFBP6
CCL3
S100B
OPC
COL2A1
RSPO2
FGF17
S100A11
IGFBP7
ADAMTS3
NRG3
NRG1
PLXDC2
EYS
LGALS1
EFEMP2
Microglia
S100A4
S100A6
S100A10
ANXA1
IGFBPL1
NRG1
NRG3
PLXNA4
ADAMTS17
LTBP1
Endothelial
RSPO2
COL2A1
RSPO1
CXCL14
FST
NRG3
PLXDC2
NRG1
THSD4
EYS
Relative expression
-2
-1
0
1
2
Late-first trimester
Early-second trimester
Late-second trimester

**Supplementary Fig. 4: Temporal-specific matrisome signatures in each cell type**

**a** Bar graph displaying the number of donors per cell type across age groups.

**b** Heatmap displaying the expression levels of the top matrisome genes across the late first trimester, early second trimester, and mid-second trimester in different cell types. Each row represents a gene, and each column corresponds to a developmental period, highlighting cell type-specific marker genes (ranked by $\log_2$ fold-change, adjusted $p$-value < 0.05). Differential expression was assessed using DESeq2 (negative binomial GLM, two-sided Wald test) with Benjamini–Hochberg FDR correction; effect size is $\log_2$ fold change with 95% Wald confidence intervals ($n$ = as specified in **Supplementary Fig. 1d**).

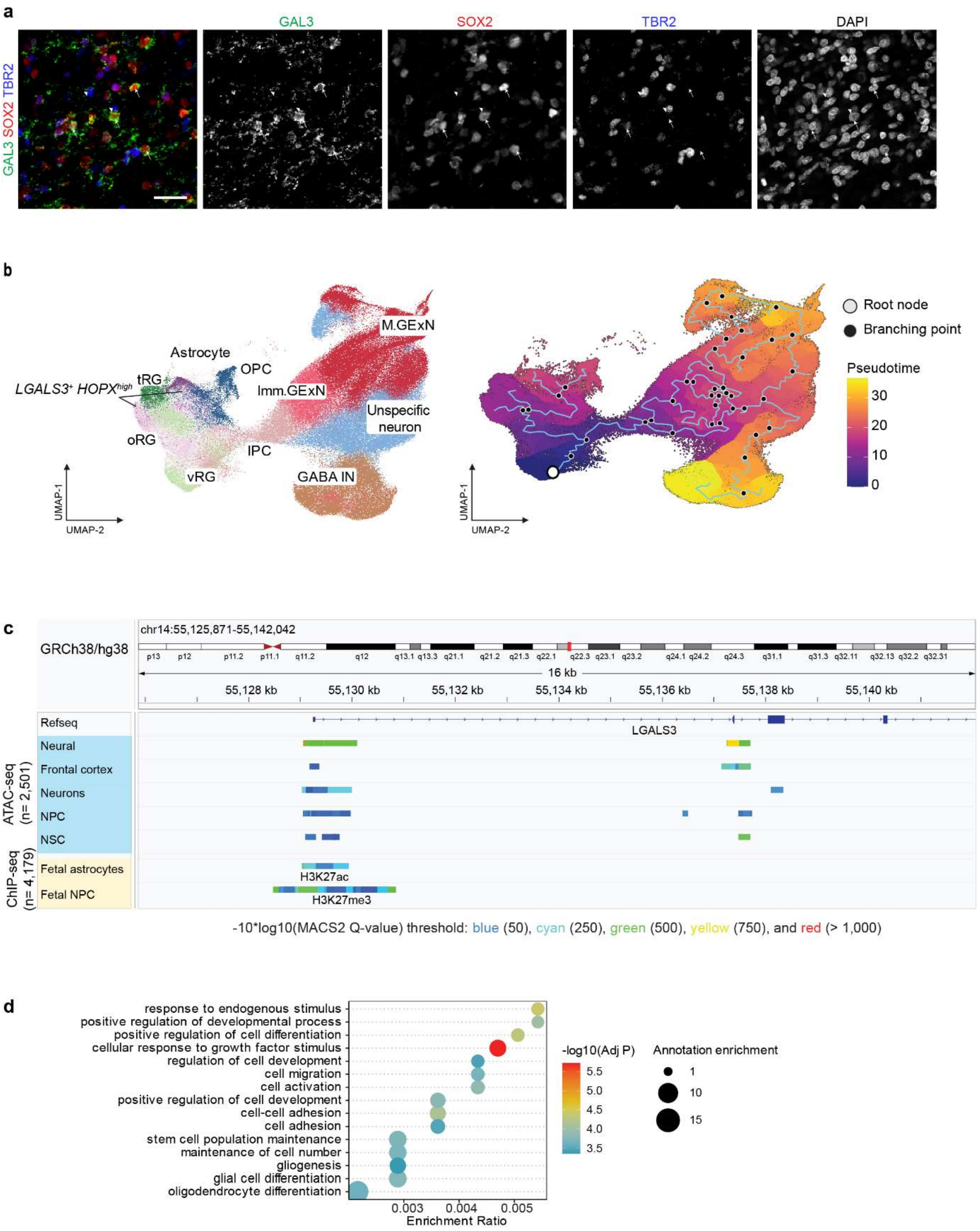


**Supplementary Fig. 5: Characterization of *LGALS3⁺ HOPX^high* cells and genomic regulatory landscape of *LGALS3***

**a** Immunofluorescence staining of SOX2, TBR2, GAL3 and DAPI in the fetal prefrontal cortex oSVZ at GW17. Scale bars: 20μm. The experiment was repeated 3 times independently with similar results.

**b** UMAP visualizations of scRNA-seq meta-data in the developing cortex. Left: UMAP showing cell type annotations and highlighting and *LGALS3*$^{+}$ *HOPX*$^{high}$ cells. Right: UMAP illustrating the developmental trajectory inferred using pseudotime analysis. Pseudotime, computed from gene expression profiles of each cluster in the meta-data, is represented by a color scale. The root node (vRG) and key branching points of the developmental trajectory are indicated.

**c** Chromatin accessibility and histone modification profiles at the *LGALS3* locus. Genomic tracks display chromatin accessibility (ATAC-seq) and histone modifications (H3K27ac, H3K27me3) across different cell types. The tracks represent signal peaks from 2,501 ATAC-seq datasets and 4,179 ChIP-seq datasets at chr14:55,125,871-55,142,042 (16 kb). Signal strength is represented by statistical significance and color intensity based on MACS2 Q-values (-10*log10 transformed), with thresholds indicated by blue (50), cyan (250), green (500), yellow (750), and red (>1,000).

**d** Gene Ontology (GO) term analysis of Group 1 transcription activators regulating *LGALS3*. The x-axis represents the enrichment score, dot size indicates the number of genes in each annotation, and the -$\log_{10}$ adjusted *p*-value is represented by the color scale.

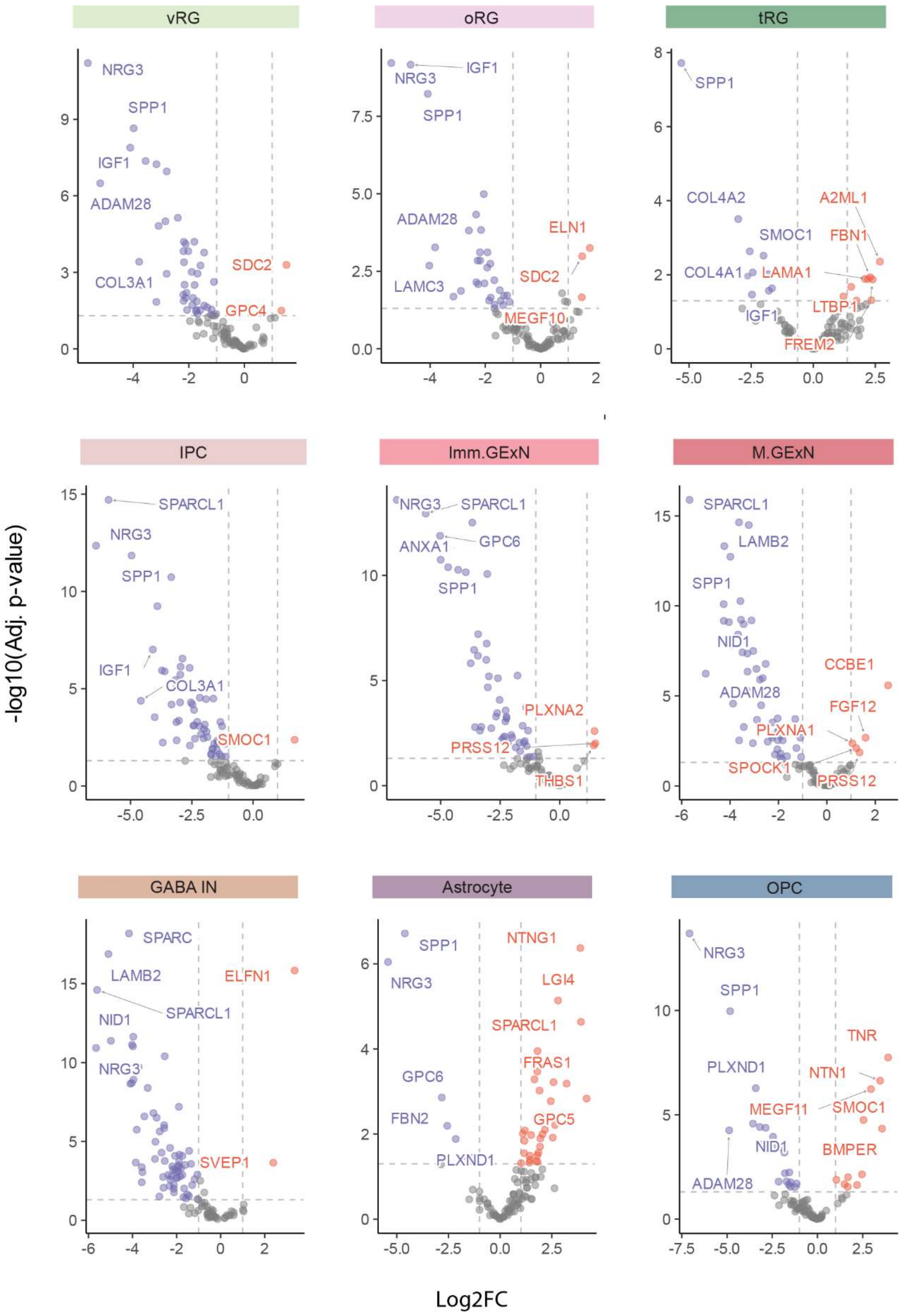


**Supplementary Fig. 6: Differentially expressed matrisome genes associated with NDDs**

Volcano plot illustrating differentially expressed matrisome genes in each cell type. The top five significantly upregulated and downregulated NDD associated matrisome genes are labeled (ranked by $\log_2$ fold-change >1, adjusted *p*-value < 0.05). Differential expression was assessed using DESeq2 (negative binomial GLM, two-sided Wald test) with Benjamini–Hochberg FDR correction; effect size is $\log_2$ fold change with 95% Wald confidence intervals (*n* = as specified in **Supplementary Fig. 1d**).
