# Supplementary material for: Deciphering cell-type-and temporally specific matrisome expression signatures in human cortical development and neurodevelopmental disorders via scRNA-seq meta-analysis
Source: Nat Commun. 2025 Nov 11;16:9907. doi: 10.1038/s41467-025-64381-3 (PMC12606316; doi:10.1038/s41467-025-64381-3)
Supplement: Supplementary file 2 — Description of Additional Supplementary Files [file 41467_2025_64381_MOESM2_ESM.pdf]

Description of Additional Supplementary Files

Supplementary Data 1. List of Cell-Type Marker Genes Used for Cell Annotation. A list of canonical positive and negative marker genes used to annotate cell type in meta-data.

Supplementary Data 2. scType algorithm score and ranking score. Scores and rankings produced by the scType algorithm for each cluster. Each cluster was annotated with the top-ranked cell type.

Supplementary Data 3. List of cell-type-specific matrisome marker genes. Differentially expressed matrisome genes in each cell type. Differential expression was tested using DESeq2 (negative binomial GLM, two-sided Wald test) with Benjamini–Hochberg FDR correction. Effect size is reported as $\log_2$ fold change with 95% Wald confidence intervals. Exact adjusted p-values are provided. Sample sizes (n) are specified in Supplementary Fig. 1d.

Supplementary Data 4. List of differentially expressed matrisome genes in NSC subtype. Differentially expressed matrisome genes in each NSC subtype. Differential expression was tested using DESeq2 (negative binomial GLM, two-sided Wald test) with Benjamini–Hochberg FDR correction. Effect sizes are reported as $\log_2$ fold changes with 95% Wald confidence intervals. Exact adjusted p-values are provided. Sample sizes (n) are specified in Supplementary Fig. 1d.

Supplementary Data 5. List of matrisome genes with temporal dynamics in each cell type. A list of temporally regulated matrisome genes in each cell type with r statistic value and p-value. Summary table is provided to present an overview of number of temporally regulated genes in each cell type. See Methods for statistical test methods.

Supplementary Data 6. List of temporally regulated matrisome genes. Differentially expressed matrisome genes at late-first (first), early-second (early) and late-second (late) in each cell type. Differential expression was tested with DESeq2 (negative binomial GLM, two-sided Wald test) with Benjamini–Hochberg FDR correction; effect size is $\log_2$ fold change with 95% Wald confidence intervals; exact adjusted p values are reported; n = specified in Supplementary Fig. 1d.

Supplementary Data 7. List of differentially expressed matrisome genes of the cells in neurogenic lineage. Differentially expressed matrisome genes of the cells in neurogenic lineage (NSC, IPC and GExN). Differential expression was tested with DESeq2 (negative binomial GLM, two-sided Wald test) with Benjamini–Hochberg FDR correction; effect size is $\log_2$ fold change with 95% Wald confidence intervals; exact adjusted p values are reported; n = specified in Supplementary Fig. 1d.

Supplementary Data 8. A list of potential LGALS3 regulators. Regulatory relationships were inferred using the IReNA workflow with pseudotime-ordered scRNA-seq data. Genes were grouped by K-means clustering of pseudotime-ordered expression profiles, with each group representing sets of genes sharing similar temporal expression patterns. Correlations ($|r| > 0.6$) were combined with motif-binding evidence (TRANSFAC, motifmatchr v1.28.0, GRCh38) to identify transcription factor–target gene interactions.

Supplementary Data 9. List of differentially expressed matrisome genes of the cells in macroglial lineage. Differentially expressed matrisome genes of the cells in macroglial lineage (NSC, IPC and GExN). Differential expression was tested with DESeq2 (negative binomial GLM, two-sided Wald test) with Benjamini–Hochberg FDR correction; effect size is $\log_2$ fold change with 95% Wald confidence intervals; exact adjusted p values are reported; n = specified in Supplementary Fig. 1d.

Supplementary Data 10. A list of cell-type specific matrisome marker genes associated with NDDs. A list of matrisome markers associated with NDD in each cell type. A value of 1 indicates association of the corresponding gene with an NDD phenotype, whereas 0 indicates no association. Differential expression was tested using DESeq2 (negative binomial GLM, two-sided Wald test) with Benjamini–Hochberg FDR correction. Effect sizes are reported as $\log_2$ fold changes with 95% Wald confidence intervals. Exact adjusted p-values are provided, and sample sizes (n) are specified in Supplementary Fig. 1d.

Supplementary Data 11. Differentially expressed NDD-associated matrisome genes in each cell type. Differential expression was tested with DESeq2 (negative binomial GLM, two-sided Wald test) with Benjamini–Hochberg FDR correction; effect size is $\log_2$ fold change with 95% Wald confidence intervals; exact adjusted p values are reported; n = specified in Supplementary Fig. 1d.

Supplementary Data 12. List of temporlaly regulated NDD associated matrisome genes. Differentially expressed NDD associated matrisome genes at late-first (first), early-second (early) and late-second (late) in each cell type. Differential expression was tested with DESeq2 (negative binomial GLM, two-sided Wald test) with Benjamini–Hochberg FDR correction; effect size is $\log_2$ fold change with 95% Wald confidence intervals; exact adjusted p values are reported; n = specified in Supplementary Fig. 1d.

Supplementary Data 13. Published year, sample number, gestational weeks, sex, and brain region of donors; the number of cells before and after quality control (QC); and the DOI of each publication are indicated. Note that sex information was reported in only one study (Cameron et al.) in the original publication.

Supplementary Data 14. List of antibodies used. Host species, manufacturer, catalog number, and dilution factor for the antibodies used in immunohistofluorescence in this study are indicated.
