# Supplementary material for: Deciphering cell-type-and temporally specific matrisome expression signatures in human cortical development and neurodevelopmental disorders via scRNA-seq meta-analysis
Source: Nat Commun. 2025 Nov 11;16:9907. doi: 10.1038/s41467-025-64381-3 (PMC12606316; doi:10.1038/s41467-025-64381-3)
Supplement: Supplementary file 17 — Reporting Summary [file 41467_2025_64381_MOESM17_ESM.pdf]

nature portfolio

Corresponding author(s): Eunchai Kang

Last updated by author(s): 19/02/2025

Reporting Summary

Nature Portfolio wishes to improve the reproducibility of the work that we publish. This form provides structure for consistency and transparency in reporting. For further information on Nature Portfolio policies, see our Editorial Policies and the Editorial Policy Checklist.

Statistics

For all statistical analyses, confirm that the following items are present in the figure legend, table legend, main text, or Methods section.

n/a	Confirmed	
☐	☒	The exact sample size (*n*) for each experimental group/condition, given as a discrete number and unit of measurement
☐	☒	A statement on whether measurements were taken from distinct samples or whether the same sample was measured repeatedly
☐	☒	The statistical test(s) used AND whether they are one- or two-sided<br>*Only common tests should be described solely by name; describe more complex techniques in the Methods section.*
☐	☒	A description of all covariates tested
☐	☒	A description of any assumptions or corrections, such as tests of normality and adjustment for multiple comparisons
☐	☒	A full description of the statistical parameters including central tendency (e.g. means) or other basic estimates (e.g. regression coefficient) AND variation (e.g. standard deviation) or associated estimates of uncertainty (e.g. confidence intervals)
☐	☒	For null hypothesis testing, the test statistic (e.g. *F*, *t*, *r*) with confidence intervals, effect sizes, degrees of freedom and *P* value noted<br>*Give P values as exact values whenever suitable.*
☒	☐	For Bayesian analysis, information on the choice of priors and Markov chain Monte Carlo settings
☐	☒	For hierarchical and complex designs, identification of the appropriate level for tests and full reporting of outcomes
☐	☒	Estimates of effect sizes (e.g. Cohen's *d*, Pearson's *r*), indicating how they were calculated

Our web collection on statistics for biologists contains articles on many of the points above.

Software and code

Policy information about availability of computer code

Data collection	Single nucleus (sn) and single cell (sc)RNA-seq data were collected from six independent brain tissue datasets. Each dataset was individually processed and the groups of cortical cells were isolated, and integrated into the final object. Preprocessing and integration were performed using Seurat (v5.1.0, R: v4.2.2). Peripheral blood mononuclear cell (PBMC) datasets were retrieved from SeuratObject (v5.0.2). Assay for transposase-accessible chromatin (ATAC) and Chromatin immunoprecipitation (ChIP) -seq datasets were obtained from ChIP Atlas and visualized on igv (v2.13.1). Motif datasets were retrieved from TRANSFAC database. Immunofluorescence image data was collected using Zen in Carl Zeiss LSM880
Data analysis	Downstream analyses, including temporal gene expression analysis and differential gene expression analysis, were performed on R (v4.2.2) using in-house built custom functions combining with DESeq2 (v1.44.0). Weighted gene coexpression network analysis (WGCNA), cell-to-cell interaction and pseudotime analyses were performed using WGCNA (v1.73), CellChat (v2.1.2) and monocle3 (v1.3.7). Other R packages used in this study include: rstatix (v0.7.2), ggpubr (v0.6.0), IReNA, ComplexHeatmap (v2.20.0), ggplot2 (v3.5.1) and motifmatchr (v1.28.0). Gene ontology analysis was performed using ToppGene. Immunofluorescence images were analyzed using Fiji ImageJ software.

For manuscripts utilizing custom algorithms or software that are central to the research but not yet described in published literature, software must be made available to editors and reviewers. We strongly encourage code deposition in a community repository (e.g. GitHub). See the Nature Portfolio guidelines for submitting code & software for further information.

Data

Policy information about availability of data

All manuscripts must include a data availability statement. This statement should provide the following information, where applicable:

- Accession codes, unique identifiers, or web links for publicly available datasets
- A description of any restrictions on data availability
- For clinical datasets or third party data, please ensure that the statement adheres to our policy

All data are available in the main text, the supplementary information and Source data file.
All code used in this study is archived and available at Zenodo: https://doi.org/10.5281/zenodo.16811863, which corresponds to the GitHub repository at https://github.com/KANGBERGLAB/matrisome-meta-analysis.

Research involving human participants, their data, or biological material

Policy information about studies with human participants or human data. See also policy information about sex, gender (identity/presentation), and sexual orientation and race, ethnicity and racism.

Reporting on sex and gender	n/a
Reporting on race, ethnicity, or other socially relevant groupings	n/a
Population characteristics	n/a
Recruitment	Women seeking elective medical termination of pregnancy were recruited with written informed consent by NHS Grampian research nurses, who operate independently of the research team. Normal pregnancies were confirmed by ultrasound, and participants included women over 16 years of age who speak English, with gestational ages ranging from 7 to 20 weeks. Grossly abnormal fetuses were excluded, and women experiencing significant distress were not approached.
Ethics oversight	Fetal tissues from elective terminations of normally progressing pregnancies were collected as part of the Scottish Advanced Fetal Research (SAFeR) study (NCT04613583)[1]. The collection process was approved by one of the 12 Scottish National Health Service Research Ethics Committees (REC 15/NS/0123) and complies with the Declaration of Helsinki guidelines

Note that full information on the approval of the study protocol must also be provided in the manuscript.

Field-specific reporting

Please select the one below that is the best fit for your research. If you are not sure, read the appropriate sections before making your selection.

☒ Life sciences ☐ Behavioural & social sciences ☐ Ecological, evolutionary & environmental sciences

For a reference copy of the document with all sections, see nature.com/documents/nr-reporting-summary-flat.pdf

Life sciences study design

All studies must disclose on these points even when the disclosure is negative.

Sample size	For the meta-analysis, sample sizes were determined by the number of samples available in the included datasets. For immunohistochemistry, sample sizes were limited by human tissue availability; at least three biological replicates were used for quantification.
Data exclusions	Before integration, individual subjects with markedly low or high nuclei number in relation to the remaining dataset were removed. For fetal tissue study, grossly abnormal fetuses were excluded, and women experiencing significant distress were not approached.
Replication	Samples used in this study are fetal prefrontal cortices from gestation weeks(GW); GW9 (Male), GW11 (Male), GW12 (Female), GW14 (Male), GW15 (female), two GW16 (both female) and two GW17 (both male). Immunoflueorescence staining and imaging for each sample were performed two to three times for experimental reproducibility of the qualitative data. For the quantitative data, the mean counts of three biological replicates for HOPX/Galectin-3 staining and four biological replicates for OLIG2 and S100β staining across GW 15-17 were calculated. Thereafter, the statistical analysis of mean count differences (pairwise t-test) was performed.
Randomization	Randomization was not relevant to this study. Groups (case and control) were determined independently by Nation Health Service (NHS) Grampian research nurses based on clinical characteristics of donors. We have used all the samples available to us that fulfilled the diagnostic requirements and selected only for gestation weeks to use in our experiments.
Blinding	For the automatic probe quantification, object-based colocalization analysis and semi-supervised cell count were performed using Colocalization Image Creator and Colocalization Cell Counter plugins on imageJ. Number of probe colocalization was calculated by the plugins.

Reporting for specific materials, systems and methods

We require information from authors about some types of materials, experimental systems and methods used in many studies. Here, indicate whether each material, system or method listed is relevant to your study. If you are not sure if a list item applies to your research, read the appropriate section before selecting a response.

Materials & experimental systems

n/a	Involved in the study
☐	☒ Antibodies
☒	☐ Eukaryotic cell lines
☒	☐ Palaeontology and archaeology
☒	☐ Animals and other organisms
☒	☐ Clinical data
☒	☐ Dual use research of concern
☒	☐ Plants

Methods

n/a	Involved in the study
☒	☐ ChIP-seq
☒	☐ Flow cytometry
☒	☐ MRI-based neuroimaging

Antibodies

Antibodies used

Antibodies used in this study are: SOX2 (1:200, Abcam, Ab79351), EOMES (1:400, Sigma, HPA-028896), LGALS3 (1:200, Invitrogen, 14-5301-82), S100β (1:400, Merk, S2532), OLIG2 (1:500, Merk, AB9610) and HOPX (1:100, rabbit Proteintech, 11419-1-AP or mouse Santa Cruz sc-398703). Secondary antibodies used in this study are: donkey anti-mouse (1:500, Cy2, Jackson ImmunoResearch, 715-225-150), donkey anti-rabbit (1:400, Cy3, Jackson ImmunoResearch, 711-167-003), goat anti-rat (1:1,000, Alexa Flour 647, Invitrogen, A-11015), donkey anti-mouse (1:400, Cy5, Jackson ImmunoResearch, 715-175-150), and donkey anti-rabbit (1:200, Cy2, Jackson ImmunoResearch, 711-225-152).

Validation

Validations were conducted based on the manufacturer's websites or previously published work.

Plants

Seed stocks

n/a

Novel plant genotypes

n/a

Authentication

n/a
